# How to control synaptic autophagy from the neuronal soma

**DOI:** 10.1038/s44318-025-00535-9

**Published:** 2025-08-21

**Authors:** Sheng Huang, Stephan J Sigrist

**Affiliations:** 1 Institute for Biology/Genetics, Freie Universität Berlin, Berlin, Germany; 2 NeuroCure Cluster of Excellence, Charité Universitätsmedizin, Berlin, Germany

**Keywords:** Autophagy & Cell Death, Membranes & Trafficking, Neuroscience

## Abstract

New research in *The EMBO Journal* shows fly Rab39 to mediate long-range control of distal synaptic autophagy from the soma.

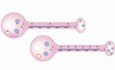

## Teaser

Autophagy dysregulation at the neural synapse is a hallmark of Parkinson’s disease and has diverse molecular origins. New research in *The EMBO Journal* investigates the role of *Drosophila* Rab39, and uncovers a system for compartmentalized regulation of synaptic autophagy, with implications for our understanding of dysregulated synaptic proteostasis as observed in neurodegenerative diseases.

Neurons are highly polarized, post-mitotic cells with expansive architecture and distal synaptic compartments. Maintenance of proteostasis at the synapse is critical for the metabolically demanding neurotransmission. It relies on tightly regulated autophagic clearance of defective synaptic components and organelles. Loss of synaptic proteostasis as a consequence of dysregulated autophagy is one of the major drivers of brain aging and neurodegeneration (Karpova et al, 2025).

Parkinson’s disease (PD) is a genetically influenced, progressive neurodegenerative disorder (Lim and Klein, 2024). While PD-associated mutations per se affect diverse cellular processes, genetic modeling of PD in animal models converges on dysregulated autophagy. Importantly, autophagy dysregulation has also been observed in the brains of PD patients (Hou et al, 2020). Prior to the onset of pathological symptoms and ultimately neurodegeneration, PD is believed to be associated with subtle synaptic and neurotransmission defects (Schirinzi et al, 2016). Therefore, the understanding of how distinct PD-linked mutations influence synaptic autophagy may uncover a common pathogenic network.

Autophagic flux at synapses depends on both the anterograde delivery of autophagic machinery and the retrograde transport of cargo-containing autophagosomes back to the soma (Karpova et al, 2025). At distal presynapses, autophagosome formation requires LRRK2-mediated phosphorylation of the endocytic protein EndophilinA (EndoA) (Soukup et al, 2016). PD variants of LRRK2 or EndoA disrupt this process and promote neurodegeneration (Soukup et al, 2016).

**Figure 1:**
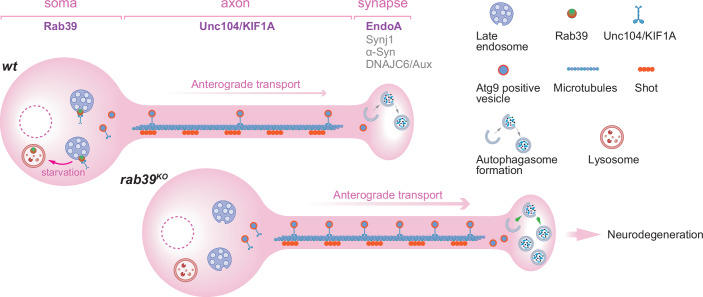
Compartmentalized cellular mechanisms of Parkinson’s disease. Parkinson’s disease (PD)-linked genes regulate synaptic autophagy through compartmentalized neuronal mechanisms. At presynaptic terminals, autophagy is locally controlled by PD-associated proteins, including EndophilinA, Synaptojanin-1, DNAJC6/Auxilin, and α-Synuclein. Rab39, localized to somatic late endosomes, gates the axonal transport of Atg9-positive vesicles via Unc104/KIF1a and Shot to support synaptic autophagosome formation. Under starvation, Rab39 shifts toward late lysosomes, likely promoting Atg9 vesicle delivery. Loss of Rab39 increases synaptic autophagy and causes neurodegeneration. Parts of this figure were created with BioRender.com.

In this issue of *The EMBO Journal*, Kilic et al (2025) combine a set of classical approaches to investigate the common molecular mechanisms of distinct *Drosophila* PD models. Building on their previous studies on synaptic autophagy activated by LRRK2-mediated EndoA phosphorylation (Bademosi et al, 2023; Soukup et al, 2016), they use electroretinograms (ERGs) as a sensitive readout of neurotransmission in a large-scale unbiased screen for genetic modifiers of the severe ERG defects in flies expressing a phospho-mimetic EndoA mutant (*endoA*^*S75D*^), which mimics constitutive LRRK2 activation. Using ethyl methanesulfonate (EMS)-induced random mutagenesis for ERG screening followed by whole-genome sequencing, they identify the microtubule-binding cytoskeleton protein Shortstop (Shot) as a key interactor of EndoA (Fig. 1). These experiments advocate classical approaches such as ERG recordings and EMS mutagenesis in tackling complex neurodegenerative mechanisms.

Notably, several of the recovered modifiers also suppress phenotypes in distinct PD models, including gene knockouts of *rab39*, *synaptojanin*, and *lrrk* (Kilic et al, 2025). These findings are exciting, as they point to a potential shared molecular machinery that links genetically diverse PD mutations through their effects on synaptic autophagy (Fig. 1). Strikingly, the knockout of *Drosophila rab39*, homologous to human PD-associated RAB39B (Gao et al, 2020), shared most of the modifiers with *endoA*^*S75D*^, suggesting a close coupling between the small GTPase Rab39 and EndoA (Fig. 1). Whether and how Rab39 collaborates with EndoA to control synaptic autophagy thus becomes a central mechanistic question emerging from this study.

Using the highly accessible *Drosophila* neuromuscular junction (NMJ), the authors then further investigate the role of Rab39 in presynaptic autophagy through in-depth genetic manipulations. They show that a complete *rab39* gene knockout (*rab39*^*KO*^), a GTPase-deficient mutant (*rab39*^*S23N*^), and a knockin of human PD pathogenic variant (*RAB39B*^*T168K*^) all resulted in excessive build-up of synaptic autophagosomes evidenced by an increased number of Atg8-positive puncta at NMJ terminals (Kilic et al, 2025). Importantly, this phenotype was accompanied by increased axonal transport and synaptic incorporation of Atg9-positive vesicles (Fig. 1), which are essential for autophagosome formation (Mari et al, 2010), while transport and accumulation of synaptic vesicles and mitochondrial components did not change (Kilic et al, 2025).

The authors were puzzled by a spatial paradox here: Rab39 itself is restricted to the soma, raising the question of how a somatically localized factor exerts long-range control over distal synaptic degradation (Fig. 1). In this regard, the shared modifiers identified in the EndoA-based genetic screen provided further mechanistic insight. Several components of the cytoskeleton and vesicle transport machinery, including the kinesin motor Unc104/KIF1a, were found to suppress the synaptic autophagosome accumulation observed in *rab39*^*KO*^ mutants (Kilic et al, 2025). These findings support a model in which Rab39 sets a “somatic bottleneck” on Atg9 vesicle export: in its absence, this restraint is lifted, allowing increased anterograde delivery of Atg9-positive vesicles to presynaptic terminals, thereby fueling excessive EndoA-dependent synaptic autophagy (Fig. 1).

These results position Rab39 as a regulator of basal autophagy in controlling the steady-state supply of degradative capacity to synapses from a distance (Fig. 1). Furthermore, they contribute to an important conceptual shift: Rab39 may not only act as a static brake but could also serve as a dynamic gate for distal autophagy in response to cellular demand. Under starvation, Rab39 partially shifts from endosomes to lysosomes in the soma, suggesting that its localization and probably its activity respond to metabolic state (Fig. 1). This raises the possibility that Rab39 toggles between vesicle retention and release, scaling the flow of Atg9-positive vesicles to match cellular need. Such dynamic regulation would allow neurons to modulate presynaptic autophagy in response to acute stress or activity, maintaining proteostasis where it is most needed.

Yet, several open questions remain. The conclusion that Rab39 functions exclusively in the soma would benefit from further validation in the future, ideally using quantitative localization and perhaps higher-resolution microscopy. Furthermore, it remains unclear how Rab39 specifically gates axonal delivery of Atg9-positive vesicles in the soma. Rab GTPases are key regulators of membrane trafficking (Wandinger-Ness and Zerial, 2014). Identifying direct regulators of Rab39 GTPase activity in the future will provide insights into Rab39 cargo selection and axonal transport entry gating.

Despite these unresolved issues, the study makes an important conceptual advance. It reframes synaptic autophagy not simply as a local response to presynaptic metabolic need, but as a process governed, at least in part, by logistic supplying of autophagic machinery originated in the soma (Fig. 1). Rab39 acts as a kind of “brake” on this system: in its absence, the soma promotes outflow of autophagic machinery to the presynapse, thereby triggering a pathological synaptic proteome and leading to neurodegeneration (Kilic et al, 2025). Restoring Rab39 function or mimicking its gatekeeping role could represent a new avenue for neuroprotective therapies.

This compartmentalized soma-to-synapse regulatory axis may be particularly relevant in the aging brain, where neurons are especially vulnerable to proteostatic imbalance (Karpova et al, 2025). In PD, the convergence of multiple PD-linked proteins suggests that dysregulated axonal trafficking of autophagic vesicles could be a unifying mechanism of synaptic vulnerability (Fig. 1). In this sense, measuring axonal anterograde transport of Atg9-positive vesicles in scenarios of locally mediated increased synaptic autophagy, such as in *endoA*^*S75D*^ mutant, will significantly improve our systematic understanding of synaptic autophagy control.

More broadly, these findings contribute to an emerging view in which neuronal health depends on the orchestration of long-range cellular trafficking and local stress responses. By uncovering a system for soma-based cellular logistics for synaptic autophagy and proteostasis control, this study nudges the field away from the concept of unrelated PD mechanisms and toward a more comprehensive and integrated molecular network underlying the pathology of neurodegenerative diseases.
